# Surface Potential Modulation in Boronate-Functionalized Magnetic Nanoparticles Reveals Binding Interactions: Toward Magnetophoretic Capture/Quantitation of Sugars from Extracellular Matrix

**DOI:** 10.1021/acs.langmuir.3c00462

**Published:** 2023-05-26

**Authors:** Stephen Lyons, Paola Baile Pomares, Lorena Vidal, Katie McGarry, Aoife Morrin, Dermot F. Brougham

**Affiliations:** † SFI Insight Centre for Data Analytics; National Centre for Sensor Research; School of Chemical Sciences, Dublin City University, Dublin 9, Ireland; ‡ Departamento de Química Analítica, Nutrición y Bromatología, Instituto Universitario de Materiales, Universidad de Alicante, PO Box 99, 03080 Alicante, Spain; § School of Chemistry, University College Dublin, Belfield, Dublin 4, Ireland

## Abstract

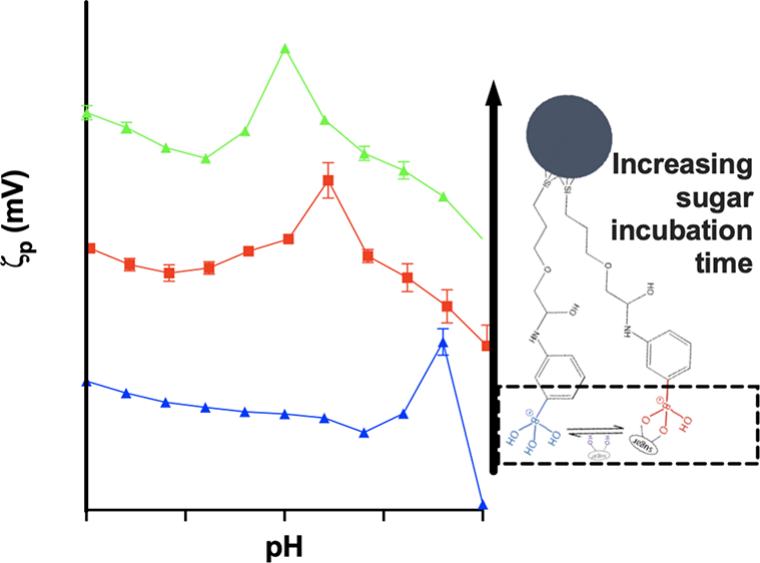

Phenylboronic acids (BAs) are important synthetic receptors that bind reversibly to cis-diols enabling their use in molecular sensing. When conjugated to magnetic iron oxide nanoparticles, BAs have potential for application in separations and enrichment. Realizing this will require a new understanding of their inherent binding modes and measurement of their binding capacity and their stability in/extractability from complex environments. In this work, 3-aminophenylboronic acid was functionalized to superparamagnetic iron oxide nanoparticles (MNPs, core diameter 8.9 nm) to provide stable aqueous suspensions of functionalized particles (BA-MNPs). The progress of sugar binding and its impact on BA-MNP colloidal stability were monitored through the pH-dependence of hydrodynamic size and zeta potential during incubation with a range of saccharides. This provided the first direct observation of boronate ionization p*K*_a_ in grafted BA, which in the absence of sugar shifted to a slightly more basic pH than free BA. On exposure to sugar solutions under MNP-limiting conditions, p*K*_a_ moved progressively to lower pH as maximum capacity was gradually attained. The p*K*_a_ shift is shown to be greater for sugars with greater BA binding affinity, and on-particle sugar exchange effects were inferred. Colloidal dispersion of BA-MNPs after binding was shown for all sugars at all pHs studied, which enabled facile magnetic extraction of glucose from agarose and cultured extracellular matrix expanded in serum-free media. Bound glucose, quantified following magnetophoretic capture, was found to be proportional to the solution glucose content under glucose-limiting conditions expected for the application. The implications for the development of MNP-immobilized ligands for selective magnetic biomarker capture and quantitation from the extracellular environment are discussed.

## Introduction

In magnetic separations, static magnetic field gradients are used to capture or concentrate target-loaded magnetic particles.^1^ Hence, particle design is often a trade-off between having sufficient magnetization per particle to provide rapid and efficient capture, as the magnetophoretic velocity is proportional to particle radius squared^1^ (favoring large particles), and having a high surface area to provide sufficient loading for detection (favoring small particles). The use of micron-scale magnetic beads for biomarker detection from biofluids including blood^2^ and serum^3^ is well-established. Commercially available Dynabeads, polymer-stabilized superparamagnetic iron oxide clusters, and similar materials provide a stable platform used in a great many examples. The maturity of these approaches is exemplified by the recent rapid roll out of mass polymerase chain reaction testing for SARS-CoV2, which uses similar beads for rapid RNA capture from buffers.^4,5^

Transport of particles in living tissue is governed by particle size, surface chemistry,^6^ and the biological barriers. For capture applications, large particles have disadvantages in the complex biopolymer network comprising tissue arising from their size,^7^ including potential exclusion or reduced extraction efficiencies due to entanglements/drag forces. Suspensions of individual superparamagnetic nanoparticles (MNPs) have possible advantages for this application. Their small size also reduces sedimentation and the likelihood of exclusion due to size,^6^ while the increased surface-to-volume can provide faster binding kinetics^8^ as well as increasing binding capacity. As the magnetic interactions are less strong their surface chemistry can also be changed to enable target binding while maintaining dispersion/preventing aggregation, which ideally should be retained following target binding. The reduced response of MNPs to applied field gradients may limit the recoverable tissue depth and reduce magnetophoretic velocity, slowing capture. However, using low-gradient (<100 T m^–1^) hand-held magnets, separations have been shown for aqueous suspensions of 12 nm MNPs,^9^ and magnetophoretic transport of 8.9 nm MNPs through the biopolymer agarose has been demonstrated.^1^ Hence, there are possibilities for magnetic capture from tissues, in particular from subdermal layers through the use of wearable magnetic patches that could also house sensing capabilities. In recent years, technologies underpinning wearable, on-skin chemical and biosensors have matured,^10^ and real-life application is increasingly close. Reported in vivo accounts include magnetic capture using antibody-labeled micron-sized beads from synovial fluid.^11^ The chemokine (C–C motif) ligand 2 (CCL2), an early marker of osteoarthritis pathogenesis, was demonstrated to be quantified in rat knee synovial fluid following a monoiodoacetate injection. The approach was based on the injection of anti-CCL2-labeled beads into the rat knee to capture the target, and following incubation, magnetic extraction was achieved using a small permanent magnet inside a catheter. Aside from this work,^11^ there are no accounts to our knowledge of quantitation following capture in vivo or evaluation of the impact of binding on colloidal stability.

**Scheme 1:**
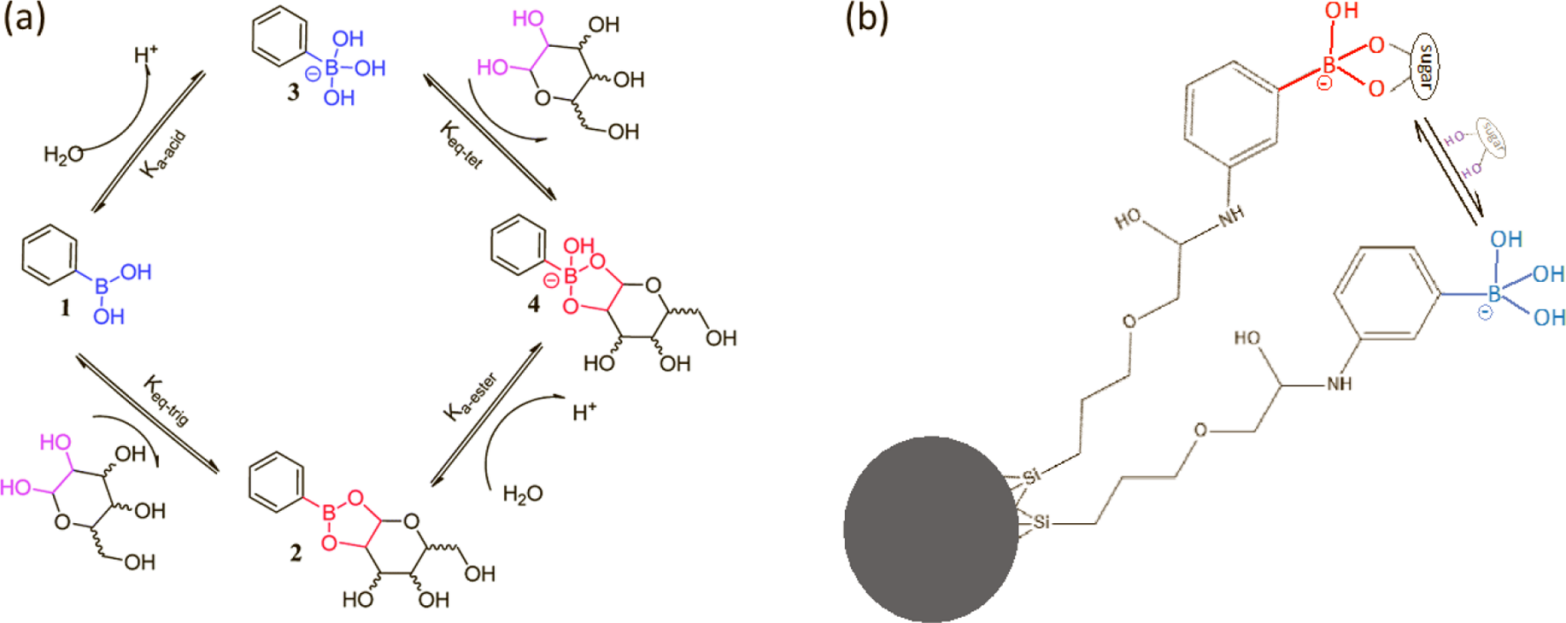
(a) Transformation of PhenylBA to Phenylboronic Ester^20^ for a *cis*-1,2-diol (Reproduced with Permission from Ref (20)., Copyright 2018, Royal Society of Chemistry); (b) Exchange of Bound Sugar Across Boronate Sites on the 3-Aminophenyl Boronic Acid Functionalized MNP Surface

Boronic acids (BAs) have been exploited in magnetic separations as they strongly bind free sugars as well as nucleic acids,^12,13^ glycoproteins,^14,15^ and glycopeptides^16^ through saccharide moieties. Binding occurs at *cis*-1,2-diol or -1,3-diol substituents by the formation of cyclic boronate esters.^17^ BA can exist in two forms: neutral trigonal (Scheme 1a, **1**) and tetrahedral anionic (Scheme 1a, **3**). The relative populations of the forms depend on the p*K*_a_ of the functional group and pH, while binding BA to particles may alter the pH dependence. Covalent binding of saccharides increases the acidity of the boronate functional group and so decreases p*K*_a_ (Scheme 1a, **2** → **4**). Under optimal pH conditions, typically between the p*K*_a_ of the BA (∼9) and the boronate ester (∼6), efficient saccharide binding occurs. In the case of phenylBAs, the p*K*_a_ of 3-aminophenylboronic acid for example is ∼8.8.^18^ Reversible covalent interactions of BAs with *cis*-diols to form cyclic esters are sufficiently strong to be of interest in magnetic separations but also in molecular sensing where they can bind saccharides at sub-mM levels, rendering BA-based sugar sensing feasible in biologically relevant scenarios.^19^

In this work colloidally stable, BA-functionalized iron oxide nanoparticles (Scheme 1b) were prepared and their potential was evaluated for magnetic separation, and subsequent quantification, of sugars from suspension and from soft-tissue environments. The iron oxide nanoparticles were functionalized with epoxy (3-glycidyloxypropyl)trimethoxysilane (GLYMO),^21^ and these groups were then further functionalized, by epoxide ring opening, with 3-aminophenylBA. The colloidal properties and stability of the resulting stable aqueous BA-MNP suspensions were evaluated. The impact of BA ionization state and of ester formation by saccharide binding on MNP stability was investigated. Zeta potential (ζp) data on BA-MNP aqueous suspensions in the absence and presence of glucose were interpreted in the context of the boronate state. Glucose binding efficiencies from the solution were measured and factors governing binding capacities are discussed. Finally, glucose binding during the magnetophoretic transport of BA-MNPs through tissue-mimetic media was investigated. The particles retained colloidal stability throughout the transit and the approach was robust, with recovery lower than for free solutions but within the usable range. The glucose bound was found to be proportional to initial glucose concentration in the matrices with sufficient sensitivity to be applicable within the clinically relevant range for dermal interstitial fluid (ISF) (4–8 mM^22^). Hence, BA-MNPs have potential for in-vivo uptake of glucose and other sugars from ISF for quantification following magnetic capture/recovery.

## Results and Discussion

**Figure 1:**
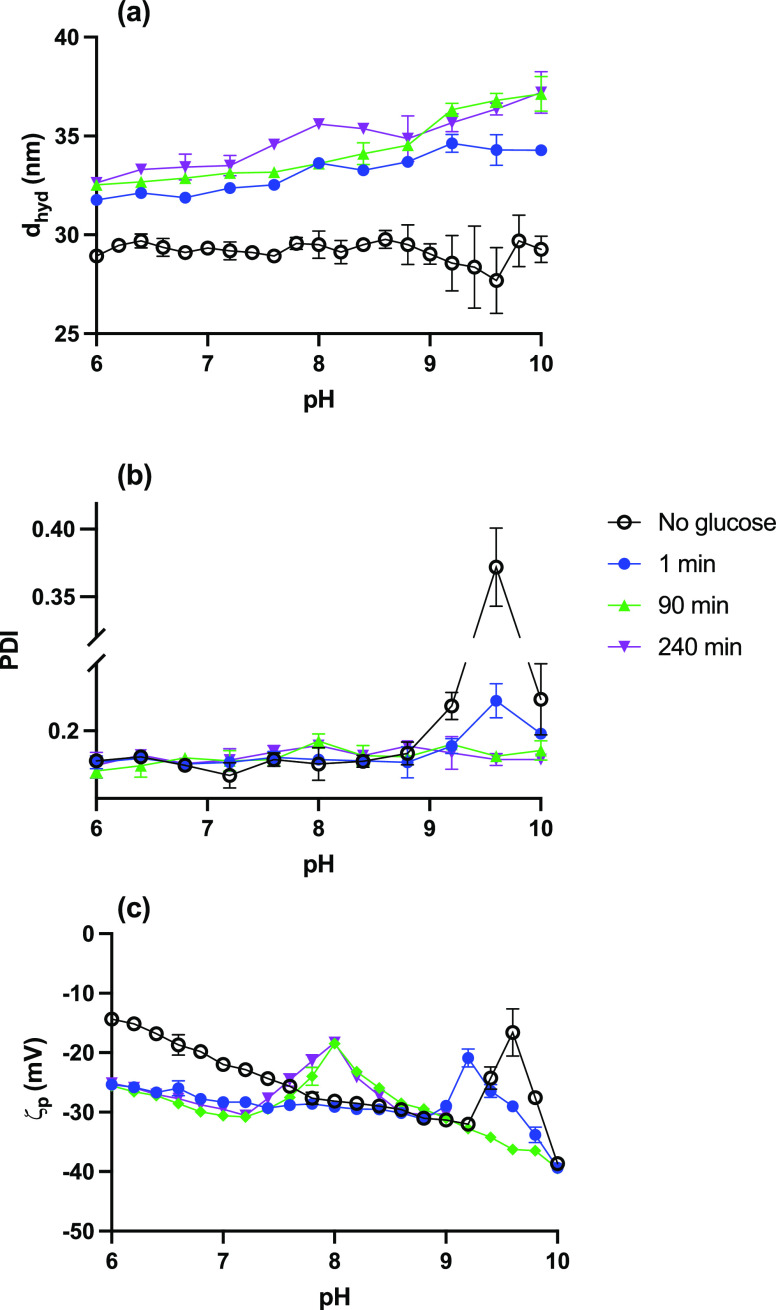
(a) *d*_hyd_; (b) PDI; and (c) ζp for 2.2 mL BA-MNP suspensions (0.40 mg particles) recorded at different pH values, before incubation with glucose and at 1, 90, and 240 min after the addition of glucose (mass 0.040 mg), see Experimental Section. There are ∼1200:1 glucose molecules:BA-MNP ensuring MNP-limiting conditions, see text. The ionic strength was 0.10 in all cases. Each data marker corresponds to the average and std. dev values measured for 4 independent suspensions. Connecting lines are added as a visual guide.

**Figure 2:**
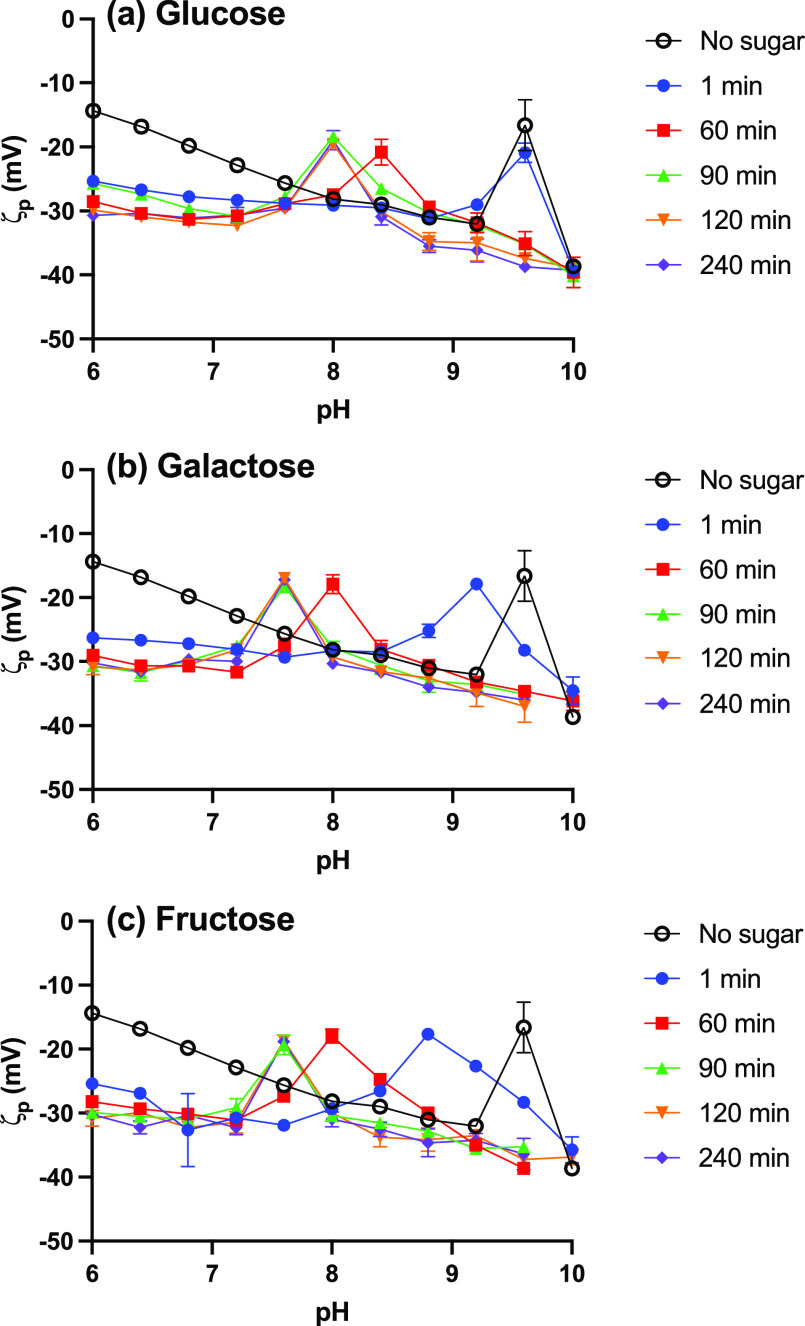
ζp measurements for 2.2 mL BA-MNP suspensions (0.40 mg particles) recorded at different pH values before (open markers) and after (closed markers) incubation with (a) glucose; (b) galactose; and (c) fructose for 1, 60, 90, 120, and 240 min after the addition of sugar as a function of pH (*n* = 4). Monosaccharide mass was 0.040 mg in all cases, giving ∼600:1 sugar molecules:BA-MNP (corresponding mass ratio 1:10) ensuring MNP-limiting conditions. Ionic strength: 0.10 in all cases.

BA-MNP suspensions were prepared as described in the Experimental Section, and their colloidal characteristics were evaluated as a function of pH in the absence of and during sugar binding. The pH of the suspensions was adjusted between 6 and 10 by adding solutions of HCl (0.10 M) and NaOH (0.10 M). To generate the data in Figures 1 and 2, individual suspensions were prepared under a given condition (pH, BA-MNP, and sugar concentration), and each was measured at the times shown and subsequently discarded or quantified. Also *n* = 4 throughout, i.e., for each point four separate suspensions were measured, one from each of four separate particle batches (four separate syntheses). While the suspensions change over several hours following exposure to sugars, the colloidal measurements take some minutes. Hence, we do not quantify the kinetics of the process. The data labeled 1 min, in particular, should be considered as “very early” in the process.

### Effect of Sugar Binding on Colloidal Properties of BA-MNP Suspensions

#### Sugar-Free BA-MNP Suspensions

The pH dependence of the hydrodynamic size, *d*_hyd_, polydispersity index (PDI), and zeta potential, ζp, of the BA-MNP suspensions are shown in Figure 1 (black markers). In the pH range ∼6 to 9, for sugar-free BA-MNP suspensions, *d*_hyd_ was observed to remain almost constant at ∼29 nm (Figure 1a black markers) and the suspensions were colloidally stable with low PDI of <0.2 (Figure 1b) indicating full particle dispersion. A progressive decrease in ζp was observed with increasing pH, from −15 mV at pH 6 down to −30 mV at pH 9 (Figure 1c), the latter corresponding to strong electrostatic stabilization. p*K*_a_ of free aminophenyl BA (Scheme 1, species **3** → **1**) is expected to be ∼8.8. So, we suggest that these changes arise from the deprotonation of a fraction of residual surface iron-oxide, which indicates that the GLYMO layer is not complete, see below.

In a narrow pH range from ∼9.0 to ∼9.6; (i) PDI progressively increased demonstrating some colloidal disruption; (ii) *d*_hyd_ decreased, which is surprising, but given the higher PDI values we suggest the size from cumulants analysis is less reliable, and; (iii) ζp increased sharply, from −31 to −15 mV, indicating that the colloidal disruption is due to reduced electrostatic stabilization. A maximum zeta potential value, ζp(max), was observed at pH 9.6 which coincides with the maximum PDI/instability of the suspensions (Figure 1a,b). In a similar range, pH 9.2–9.8, the zeta analysis is also less stable showing bimodal distributions with similar ζp values for each mode, Table S1. Hence, while the zeta analysis reflects a reduction in surface negative charge, we do not interpret the actual values in this pH range. At pH 10, the ζp was found to be strongly negative, probably due to the complete ionization of bound boronate, and the PDI recovered. Apparently, surface ionization of grafted BA drives colloidal interactions in this pH range, an effect most clearly seen in the ζp instability. This interpretation is confirmed by the effect of added sugars, see below.

The pH-dependence of ζp for dextran-coated MNPs functionalized with aminophenyl BA (diameter 700 nm) was reported previously, with a smooth decrease in ζp from −14 to −23 mV described between pH 8–10, but with no ζp(max) noted.^23^ Those observations are nevertheless consistent with ours; we observed a ζp decrease of similar extent (from −28 to −38 mV), and given the wider single pH unit ζp measurement intervals used in the older study it is possible that a ζp(max) feature was missed. Hence, to our knowledge, ours is the first reported observation of boronate ionization in grafted BA by light scattering techniques. It is found that (in the absence of sugar) BA decreased in acidity on grafting to the NP surface, we suggest the shift is due to the replacement of hydrogen on the amino group by the alkyl chain, see Scheme 1b (Introduction).

#### BA-MNP Suspensions with Sugars

The impact of glucose binding on the pH-dependent colloidal properties of BA-MNPs was investigated (Figure 1a–c) by incubating a series of suspensions of increasing pH (0.4 unit increments) with a fixed amount of glucose in excess of the binding capacity, i.e., under MNP-limiting conditions (which are confirmed below) for specified periods of time, see Experimental Section. The data in Figure 1 was recorded for suspensions at ∼1200 glucose molecules per MNP.

In the pH range 6–9, following incubation of BA-MNP suspensions with glucose for 1 min, *d*_hyd_ increased consistently by 1–2 nm (Figure 1a blue markers), while low PDI (Figure 1b) and high negative ζp values (Figure 1c) demonstrate full particle dispersion. The slight increase in *d*_hyd_ with pH is unlikely to be due to aggregation; given the low PDI, it is more likely to arise from greater glucose coverage. For longer incubation times, small further increases in *d*_hyd_ were observed, particularly at higher pH, and again PDI remained low, which is consistent with increasing glucose loading beyond 1 min. The process occurs over a timescale of minutes and appears to be essentially complete by 90 min.

In the pH range of 9–10, the PDI progressively improved with incubation time, approaching values typical for suspensions at pH below the ionization (Figure 1b) by 90 min. The most interesting observation is a progressive shift of ζp(max) to lower pH of 9.2 and 8.0, after 1 and 90 min glucose incubation, respectively, with only marginal further change by 240 min (Figure 1c). Additional data for 60 and 120 min incubation times, Figure S4 and Table S1, show that the final ζp(max) value is reached between 60 and 90 min. These observations confirm the assignment of the ζp(max) feature to ionization of bound BA, i.e., it coincides with an “approximate” p*K*_a_ which is shifted as expected to lower pH (increased acidity) upon ester formation.

#### Binding Affinity of BA-MNP Suspensions for Different Sugars

The effect of sugar type on surface binding was investigated by incubating BA-MNP suspensions with fixed amounts of glucose, fructose, and galactose and monitoring the development of ζp as a function of pH. Again MNP-limiting conditions were used, with ∼600 sugar molecules added per MNP in this case, and data were gathered from pH 6–10 in 0.4 unit increments (Figures 2 and S5). In all cases, a shift in the final ζp(max) to lower pH was observed on addition of sugar, and this was achieved within 90 min incubation. For galactose and fructose, the shift was to a lower pH (∼7.6) than was observed for glucose (pH ∼8.0). The response, as indicated by the pH of ζp(max) at 1 min incubation, was possibly faster for galactose (pH ∼9.2) than for glucose (no measurable shift at 1 min) and faster again for fructose (pH ∼8.8) (see also Figure S5). However, given the difficulty of the “1 min” measurement, noted above, this should be viewed with some caution. The clear greater shift in ζp(max) for similar amounts of sugar, i.e., increased bound boronate acidity, is consistent with the generally observed BA monosaccharide binding affinity of glucose < galactose < fructose^19^ (as are the, probably, faster kinetics). It might be possible to further resolve ζp(max) values at steady state for galactose and fructose with a finer sampling interval.

To summarize, the consistency in colloidal responses for different sugars achieved for *n* = 4 different MNP batches demonstrates that the outcomes are robust and reproducible. Interestingly, the final ζp(max) value obtained is the same for glucose:MNP of ∼600:1 (Figure 2) and ∼1200:1 (Figure 1), suggesting that the system is indeed MNP-limited. Under these conditions, the final ζp(max) values, in particular, reflect sugar-specific modulation of bound boronate p*K*_a_.

### Binding Capacity of BA-MNP Suspensions

As binding efficiencies vary with both ionic strength and pH,^24^ for application in biomedical sensing and enrichment protocols understanding of binding under isotonic, and ultimately physiological, conditions is needed. Furthermore, for sugar quantitation following extraction, it is important that the system is not saturated, i.e., the glucose-limiting range should be established.

#### Expected Coverage

The assumption that the system is under MNP-limiting conditions was made for the experiments shown in Figures 1 and 2. A mass of 0.040 mg saccharide was used for all these incubations, which for glucose is equivalent to 1.34 × 10^17^ molecules available for binding. The core surface area for an 8.9 nm sphere is 249 nm^2^ and we previously^1^ determined the silane grafting density to be ∼2.0 nm^–2^. Hence, the estimated number of GLYMO groups per MNP is ∼495. Given the epoxide reactivity and large excess of BA used, close to full BA functionalization might be expected, i.e., the available BA groups per MNP should be approaching 49.5. The mass of MNPs added to each vial was 0.40 mg, assuming monodisperse 8.9 nm spheres of bulk γ-Fe_2_O_3_ density (4.90 g cm^–3^), the average mass of a single MNP core can be estimated as ∼1.81 × 10^–15^ mg. So, the number of MNPs present is ∼2.21 × 10^14^ and the maximum number of BA sites available is ∼1.09 × 10^17^. Thus, for glucose, in the experiments shown in Figure 2, there were ∼600 saccharide molecules available for binding per MNP, on which there were on average ∼495 GLYMO sites. Hence, the suspensions are expected to be slightly MNP-limited. As noted above, that view is supported by observation of the same final ζp(max) value for glucose:MNP of ∼600:1 and ∼1200:1 (Figure 1).

#### Experimental Determination

A series of BA-MNP glucose incubations were performed in free PBS solution (pH 7.4, 0.10 M) in which the amounts of glucose and BA-MNPs were independently varied. Following incubation BA-MNPs were extracted from suspension using a hand-held magnet, and bound glucose was quantified by an enzymatic assay using an established calibration (Figure S3), see Experimental Section. It was found that when low glucose conditions were used that >99% of the glucose added was found in the magnetically recovered washed solid fraction. This demonstrates that (i) magnetic catching yields >99% of the suspended particles; (ii) any glucose present will bind if there are BA-MNP sites available; and (iii) the three washing cycles do not de-bind detectable saccharide so there is little, if any, nonspecifically bound glucose. When high glucose conditions were used, ∼99% of the glucose used was found in the sum of the two (magnetically caught and supernatant) fractions.

**Figure 3:**
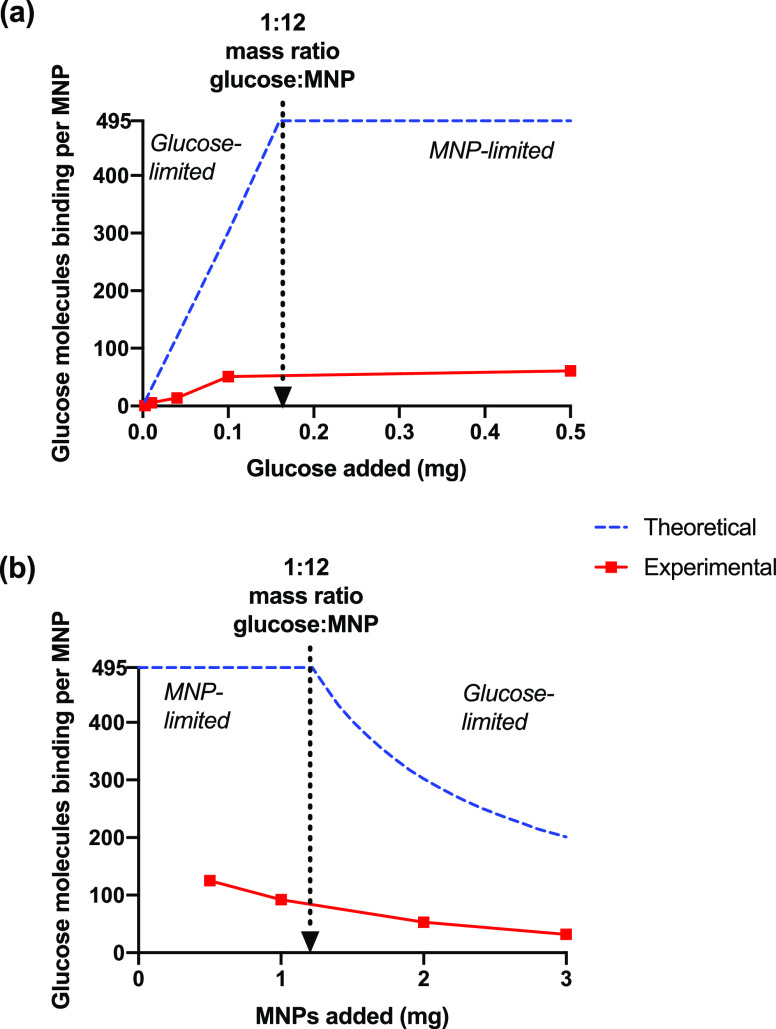
Number of glucose molecules binding to MNPs following 90 min incubation (*n* = 3 independent suspensions) in PBS (0.10 M, pH 7.4, 4.0 mL) containing; (a) BA-MNPs (2.0 mg) as a function of glucose added, and; (b) glucose (0.10 mg) as a function of BA-MNP added. The glucose-limiting range is where the mass ratio of glucose:MNP is 1:>12.

Glucose mass was first varied in a series of 90 min incubations with a fixed mass of BA-MNPs (2.0 mg). The results are shown in Figure 3a as the number of bound glucose molecules per MNP as a function of the mass of glucose added. Assuming binding is only limited by the number of available BA sites, the number bound should increase linearly with added glucose up to the theoretical limit of ∼495:1 glucose:BA-MNP (Figure 3a, blue). However, experimentally the number bound was found to be consistently lower under the conditions used. The binding per MNP was approximately linear with glucose added at low glucose concentration (glucose-limiting) which is encouraging, and an apparent limit was reached of ∼50 to 60 per MNP at <0.10 mg glucose. The limiting coverage is ∼1/10th of the expected value. This demonstrates that for the experiments shown in Figures 1 and 2, the system was actually more MNP-limited than anticipated. Incubation experiments were also performed for varying BA-MNP mass at fixed glucose mass, Figure 3b. The number bound per MNP decreased on increasing the mass of MNPs (into the glucose-limited range), approximating the expected behavior albeit at a lower binding efficiency.

The lower-than-anticipated binding, apparent from Figure 3, is unlikely to arise from electrostatic effects (free glucose is neutral at pH < 12). Also, the washing experiments show that there is no significant nonspecifically bound glucose. This suggests much lower boronate functionalization than expected as the cause of low sugar binding.

Considering the key findings. First, the pH dependence of ζp observed under MNP-limiting conditions is very revealing. The gradual shift of the ζp(max) feature to lower pH demonstrates a slow build-up of glucose coverage to what turns out to be ∼1/10th of the expected value. This is consistent with the fact that at all incubation times studied the pH dependence of ζp does not switch from positive to negative at p*K*_a_, as would be expected for high BA coverage. Second, it is interesting that as the ζp(max) feature gradually shifts to lower pH it remains 1 pH unit wide throughout (except perhaps in some cases for 1 min, the most imprecise time point). Hence, there is a single average coverage-dependent “approximate” p*K*_a_ even as the coverage slowly builds, i.e., there is no evidence for intermediate forms. We suggest that this arises from a relatively fast bound glucose averaging process. The likely possibilities are exchange with bulk glucose (slow under MNP-limiting conditions) or, more likely, bound glucose exchange between surface boronates. Despite low final sugar coverage (∼1/10th of available GLYMO functionalities), a fast surface exchange process can be envisaged, as suggested in Scheme 1b. For example, in the case of a hexagonally close packed surface ∼1/10th of the sites bearing a boronate corresponds to an average of at least one BA within the nearest or next-nearest neighbor group.

### Glucose Capture from Tissue-Mimetic Matrices

#### Magnetophoretic Capture from Gels

As a step toward application of BA-MNPs for magnetically enabled quantification of saccharides in biological tissue, we investigated capture of free glucose from agarose hydrogel (0.3% w/v), as a first mimic of tissue/extracellular matrix (ECM). MNP suspensions do not significantly penetrate agarose when they are pipetted on top. However, when a static magnet (hand-held, N52) is placed underneath, over several hours, the field gradient drags the particles, as a visible deposit, through the biphasic matrix. Hence, photographic tracking of the deposits progress, and subsequent capture of MNPs are possible. We have previously described^1^ linear transits in time (enabling extraction of an experimental velocity, *V*_exp_) for citrate-, arginine-, and PEGylated-MNPs in magnetophoretic motion through agarose gels. It was shown that *V*_exp_ decreases with increasing *d*_hyd_ (increased drag force) and with increasingly negative ζp (reduced flux through pore restrictions imposed by repulsive interactions with anionic agarose chains). Given the biphasic nature of the gels, it is expected that glucose will establish a partition equilibrium and so should be available for binding with BA-MNPs as they pass along tortuous aqueous paths. The binding of glucose to BA-MNPs in suspension is quite slow (Figure 2); hence, the slow passage of the particles through the gels may enable sufficient binding.

**Figure 4:**
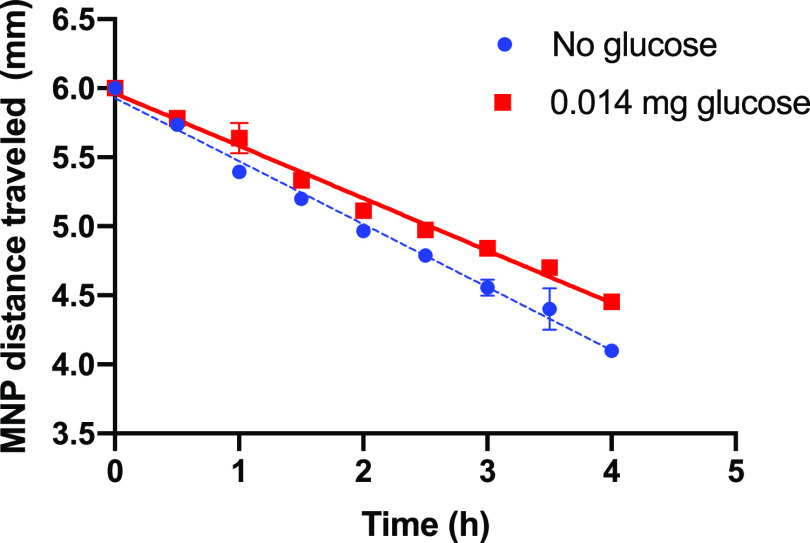
Magnetophoretic transport of 0.20 mg BA-MNPs, (added as 200 μL of 1.0 mg mL^–1^ BA-MNP stock to the top of gel) (*d*_hyd_ 29.5 nm, PDI 0.17) through 0.3% agarose/DI H_2_O (no glucose) (blue) and 0.3% agarose/DI H_2_O containing 0.014 mg glucose (red). Agarose gels (700 μL; depth 6 mm). 1:14 mass ratio glucose:MNP. Linear regression applied over full transit duration (0–4 h), *n* = 3.

Typical data showing the distance traveled by the front edge of the BA-MNP deposit during magnetophoretic transport through 6 mm-thick (see Experimental Section) agarose/H_2_O gels at close to neutral pH are shown in Figure 4. Incidentally, it is interesting that the velocity does not increase over the transit, given that the magnetic force follows an inverse square dependence with distance from the pole face. This suggests a visco-elastic response to the matrix. For the glucose-free gels, BA-MNPs rapidly attained a terminal velocity which was, as expected, maintained for the complete transit. *V*_exp_ of 0.46 mm h^–1^ was obtained by regression, a velocity that is similar to those previously reported.^1^ For glucose-loaded gels, under nominally slightly glucose-limited conditions, *V*_exp_ was slower but again linear transport was apparent, with *V*_exp_ 0.38 mm h^–1^. The change shows that glucose does bind under these conditions and that it alters the flux at the pore restrictions. Data consistent with theory was also observed in glucose-loaded agarose/ISF gels (Figure S6; *V*_exp_ 0.41 mm h^–1^) where the velocity was measured to be greater than its DI H_2_O gel equivalent. This is expected as electrostatic effects have been shown to modulate magnetophoretic velocity.^1^ After transit, the recovered glucose-exposed MNP suspensions were stable, and as expected, *d*_hyd_ increased to 33 nm (Table S2) with no change in PDI, consistent with binding but no aggregation. Hence, Figure 4 demonstrates that BA-MNPs bind glucose from agarose gels, and that magnetophoretic capture of loaded fully dispersed particles is possible. The next step is to evaluate the potential for quantitation.

#### Preliminary Evaluation in Tissue-Mimetic Models

**Figure 5:**
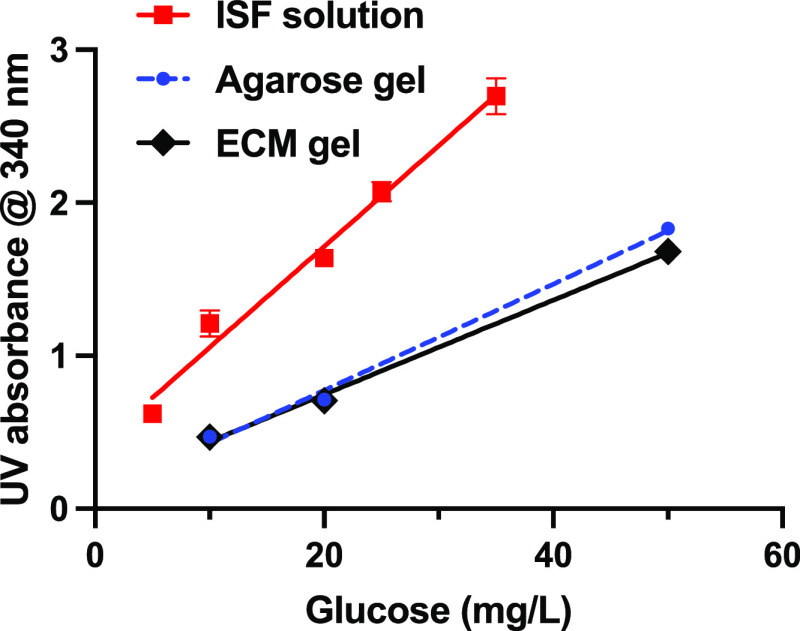
Absorbance measurements for recovered BA-MNPs following incubation in synthetic ISF solution (red), 0.3% agarose gel prepared in ISF (blue) and cultured ECM (black) as a function of glucose mass present. 0.40 mg BA-MNPs was added, as 200 μL of 2.0 mg mL^–1^ stock, to the solution or top of gel. The mass ratio range is from 1:80 to 1:12 glucose:MNP, i.e., glucose-limited conditions. The glucose concentration range was 5–50 mg L^–1^. ISF solutions were 700 μL in volume; incubation times 90 min; *n* = 4. Agarose and ECM gels were prepared from 700 μL volume solutions; depth 6 mm; recovery was after 24 h; *n* = 4.

Two types of systems were studied to assess the possibilities for quantitation following the extraction of free saccharides in tissue-mimetic scenarios: (i) rapid magnetic catching of BA-MNPs from homogeneous suspension in glucose-doped serum-free media (ISF) and (ii) slow magnetophoretic recovery of BA-MNPs following full transit through agarose or commercial ECM gels, which were made up in glucose-doped ISF. The suspensions were included to provide measures of efficient binding and recovery. MNP recovery was maximized for the gels by leaving the samples on the magnet for 24 h. In each case, the recovered material was washed and bound glucose quantified enzymatically, see Experimental Section. The glucose:MNP mass ratio was maintained at 1:>12 in all cases, to ensure nominal glucose-limited conditions. The data are shown in Figure 5.

For extraction from ISF suspension, a correlation was observed between absorbance and glucose added (slope 94.2 mg^–1^, *R*^2^ 0.98) over the range investigated (5–50 ppm). This is encouraging as it reflects greater sensitivity than needed given the range of interest for dermal ISF glucose measurements (500–2500 ppm). It suggests that there is scope for application, depending on dermal volumes, MNP masses used, etc. For extraction from the bio-mimetic gels, it is also encouraging that linear responses were measured in both cases, with reduced sensitivity, *c*. 50% of that obtained for the suspensions (agarose/ISF 50.0 mg^–1^, *R*^2^ 0.99; ECM/ISF 44.0 mg^–1^, *R*^2^ 0.995). This is expected due to lower BA-MNP recovery arising from the loss of particles at the bottom of the gels and also perhaps from within, i.e., there may be some blind paths.

Cultured ECM comprises structural proteins and proteoglycans as compared to the simpler chains in the agarose gel. So, significant differences in extraction efficiency might be expected, arising from different loadings per BA-MNP caught. For instance, differences in pore size might alter the average length of the MNP path traveled, and the glucose partitioning may differ. While this is a preliminary study, the similarity in the two gel responses (slopes) suggests that in fact particle recovery issues, which could be addressed with improved device design, predominate. Hence quantitation may prove possible for complex media, or perhaps for tissue, irrespective of the matrix composition.

## Conclusions

A robust approach to magnetic nanoparticle functionalization for sugar binding is described and the limiting ranges and particle binding capacity are identified. Under MNP-limiting conditions in suspension, sugar binding to BA-MNPs is shown to be associated with a shift in the ζp(max) feature to lower pH, arising from sugar-specific increases in boronate acidity, i.e. the maximum marks the p*K*_a_ of bound BA. The absence of a p*K*_a_ distribution during slow binding (no intermediate forms) reveals fast on-particle exchange. At all pH, the BA-MNP suspensions remain stable both with and without sugar, which is advantageous for magnetophoretic capture from complex media and tissue.

Under glucose-limiting conditions, the capture of glucose-loaded BA-MNPs from both free suspension and tissue-mimetic hydrogels was demonstrated. In all cases, bound glucose, measured following particle capture, was shown to be proportional to the concentration of free glucose used. Preliminary results show that the quantification range and extraction efficiency provide sensitivity in excess of that required for applications, so particle concentration could be further reduced while retaining glucose-limiting conditions necessary for quantitation.

For in vivo applications, porous catheter systems with retractable magnetic syringe barrels^11^ have been proposed for antibody recovery/detection. Our study suggests that a similar approach to recovery/quantification of sugars or other biomarkers, from dermal ISF or passively expressed sweat, using a cutaneous magnetic capture patch system may be feasible. Enabling factors include the observation that for BA-MNPs the extraction/quantification are, for the model hydrogels at least, not significantly influenced by gel composition. While the long magnetophoretic transits through tissue environments may be generally applicable for biomarker quantitation using more specific MNP-immobilized ligands with slow target binding kinetics.

## Experimental Section

### Reagents and Equipment

Iron(III) acetylacetonate (14024-18-1), benzyl alcohol (10051-6), (3-glycidyloxypropyl)trimethoxysilane (GLYMO) (2530-83-8), tetrahydrofuran (THF) (109-99-9), 3-aminophenylboronic acid (30418-598), agarose (9012-36-6), d-(+)-glucose (50–99-7), d-fructose (57-48-7), galactose (59-23-4), calcium chloride (10043-52-4), (4-(2-hydroxyethyl)-1-piperazineethanesulfonic acid) (HEPES) (7365-45-9), potassium chloride (7440-097), magnesium sulfate (7487-88-9), sodium chloride (7440-23-5), hydrochloric acid (HCl) (7647-01-0) monosodium phosphate (53408-95-0), saccharose (57-50-1), isopropyl alcohol (67-63-0), bovine collagen Type IV (9007-34-5), Dulbecco’s Modified Eagle Medium (DMEM) (143-74-8), extracellular matrix (ECM) gel from Engelbrecht-Holm-Swarm murine sarcoma (E1270) and glucose (HK) assay kit (GAHK20-1KT) were purchased from Sigma. Chloroform (67-66-3) was purchased from Fisher Chemical (Belgium). An N52 grade neodymium magnet (www.first4magnets.com) with dimensions 25 × 25 × 50 mm was used for magnetophoretic transport experiments. 6 × grade N40 Neodymium magnets (dimensions: 1 × 1 × 2 mm; www.first4magnets.com) were assembled as a needle structure using glue as the magnetic needle.

### MNP Synthesis

MNPs were synthesized by a method published by ourselves,^1^ which is an adaptation of the Pinna method.^25^ Briefly, the MNPs were formed by mixing iron acetylacetonate (1 g) with benzyl alcohol (20.0 mL). This mixture was placed into a G30 glass test tube and microwave digested for 3 h at 200 °C under pressure (18 bar). The resulting suspension was 50.0 mg mL^–1^ of γ-Fe_2_O_3_ MNPs. The average core diameter was previously determined to be 8.9 ± 0.8 nm by Transmission electron microscopy (TEM) for particles prepared under identical conditions.^1^ Again, as published previously,^1^ MNPs prepared using this method have been shown by us using magnetometry to be superparamagnetic at room temperature and to have saturation magnetization in the expected range.

### MNP Functionalization

BA-MNPs were functionalized using a ligand exchange process. An initial ligand exchange with GLYMO formed the silanol bonds on the MNP surface. The epoxy ring was then opened by base hydrolysis using 3-aminophenylBA to yield BA-MNPs. The chemical functionalization of the MNP is shown above (Scheme 1b). To carry out the functionalization, the iron oxide MNPs (2 mL, 1.0 mg/mL) in benzyl alcohol were added to a vial with acetone (4 mL). This caused the MNPs to precipitate out of the benzyl alcohol. The glass vial was then placed on a magnet to retain the MNPs while the benzyl alcohol/acetone mixture was removed. This step was repeated three times to ensure all benzyl alcohol was removed. GLYMO (50 μL) was dissolved in chloroform (2 mL) and added to the MNP material. This mixture was then placed on a plate shaker at 400 rpm for 24 h. THF (2 mL) was used to remove excess GLYMO by magnetic separation after agitation. GLYMO was used to modify the surface of the MNPs through the covalent attachment of functional alkoxysilanes, simultaneously providing surface epoxide groups ready for further conjugation chemistry. 3-Aminophenylboronic acid (2 mL, 2.5 mg/L in DI H2O) was added to the GLYMO-MNPs and placed on a plate shaker at 400 rpm for 5 h. KOH (50 μL, 1.0 M) was added to precipitate out the BA-MNPs. Finally, BA-MNPs were dispersed in DI H_2_O at the desired concentration. The surface chemistry is represented in Scheme 1b.

Previously, thermogravimetric analysis (TGA) confirmed functionalization with GLYMO^1^ with an estimated silane grafting density of ∼2.0 nm^–2^, and the BA surface groups in the magnetic fraction were confirmed by Fourier-transform infrared spectroscopy (Figure S1). MNP suspensions were also characterized by dynamic light scattering (DLS) using a Zetasizer Nano ZS (Malvern Instruments, UK). Experiments were performed at 25 °C and BA-MNP Z-average hydrodynamic diameter (*d*_hyd_) and polydispersity index (PDI) values from cumulants analysis are reported here (Figure S2). A PDI value below 0.2 is indicative of a full particle dispersion, once the cumulants model fits the data which was found to be the case for all suspensions. BA-MNPs were found to have *d*_hyd_ (PDI) of 29.5 nm (0.18). Zeta potential (ζp) measurements were performed at 25 °C on the Nano ZS, using the M3-PALS technology (Figure S2).

### Monosaccharide/BA-MNP Suspension Preparation, Magnetic Extraction, and Quantification

To prepare BA-MNP suspensions, 2.0 mL of DI H_2_O, or PBS, containing a specified mass of monosaccharide (typically 0.040 mg of glucose fructose or galactose) was placed in a glass vial. 200 μL BA-MNP (at typically 2.0 mg mL^–1^) suspension in DI H_2_O was added to give 2.20 mL at typical working concentrations of 0.018 and 0.082 mg mL^–1^ glucose and MNPs, respectively. The BA-MNP masses quoted are obtained for each particle batch by sacrificial drying/weighing. The mixture was agitated using a plate-shaker for a specified period of time (see below) to allow binding to proceed. 500 μL of NaCl solution (2.0 M) was then added to the mixture which was subsequently vortexed for 1 min to precipitate the BA-MNPs. The mixture was then placed on the N52 magnet for 1 min to magnetically separate the solids. The liquid was decanted and the MNPs washed three times with DI H_2_O to remove any unbound glucose. The glucose in the final washed solid fraction was then quantified enzymatically using a glucose hexokinase (HK) kit.

Briefly, 250 μL of the assay reagent containing hexokinase was added to the washed solid fraction in the vial and incubated for 30 min. The vial was then placed on the N52 magnet to remove the MNPs from the suspension. The absorbance of the resulting solution, which contains the liberated detectable saccharide product, was measured at 340 nm. Standard glucose solutions were also prepared in the range of 5–50 ppm and were quantified enzymatically in the same way. All analyses were carried out in triplicate (Figure S3).

### Agarose Gel Preparation

To prepare agarose gel (0.3% w/v), 0.06 g agarose was added to DI water, PBS solute, or synthetic interstitial fluid (2 mM CaCl_2_, 10 mM HEPES, 3.5 mM KCl, 0.7 mM MgSO_4_ 123 mM NaCl, 1.5 mM NaH_2_PO_4_)^26^ as specified. Known amounts of glucose were added to the solute where specified. The mixture was heated and stirred until the agarose had fully dissolved. Once the agarose solution was transparent, glass vials (53 × 16 × 16 mm) were filled with the agarose to a depth of 6 mm and were left to cool at room temperature for 1 h. The vials were then capped and left to solidify overnight at 4 °C.

### Cultured Extracellular Matrix Preparation

Prior to handling the cultured extracellular matrix (ECM), all workplace surfaces were washed down with isopropyl alcohol to ensure sterility. The frozen ECM solution was fully thawed at 4 °C. 700 μL of the solution was transferred to a 7 mL glass vial. Known amounts of glucose were added to the solute where specified. The vial was stored for 12 h at 4 °C to allow the release of trapped air. ECM was then incubated at 37 °C for 40 min to induce ECM polymerization by self-assembly processes. ECM gels were used for experiments within 24 h of being prepared. All glassware were washed with methanol to ensure sterility to avoid contamination.

### Glucose Extraction and Quantification from Gels Using BA-MNPs

Plastic cuvettes were modified by removing the base to form an open structure. Masking tape was temporarily applied to the base of the cuvette and gel solution (700 μL) was added to the cuvette. Gel solutions contained fixed concentrations of glucose as specified. The masking tape prevented the gel solution from leaking while it solidified. After solidifying overnight at 4 °C, gels were placed tape-edge facing down onto one of the four corners of the N52 magnet. 100 μL of a BA-MNP suspension (2.0 mg mL^–1^ in DI H_2_O) was pipetted onto the top surface of the gel to form an even layer of ∼1 mm thickness covering the entire upper gel surface (176.6 mm^2^) and the vials were capped. The BA-MNPs were observed to migrate toward the base of the vial under the influence of the magnetic field over time. The tape was removed 1 h after transport was initiated. Once the MNPs fully migrated through the gel, they were collected at the gel base on a magnetic needle. The MNPs were removed from the magnetic needle by washing vigorously with DI H_2_O. 500 μL NaCl (2 M) was then added to the washing and the solution vortexed for 1 min to precipitate the BA-MNPs. The solution was then placed on an N52 magnet to magnetically separate the BA-MNPs from the suspension. The solution was decanted and the MNPs were washed 3 times with DI H_2_O. The MNPs were then dispersed in water at a known concentration and bound glucose was quantified enzymatically using the enzyme assay kit in the same manner as described previously for the solutions.

To measure the distance traveled by the BA-MNPs in the gels over time, the vials were imaged when the BA-MNP suspension was pipetted on top of the gel and again every 30 min using a standard commercial digital camera. The distance between the top of the gel and the BA-MNP front was measured using ImageJ.

## Supporting Information Available

BA-MNP FTIR (Figure S1); size distribution profile (Figure S2); absorbance measurements for glucose solutions in PBS (Figure S3); ζp data for BA-MNP suspensions (Figure S4); tabulated ζp data for 2.2 mL BA-MNP suspensions (Table S1); ζp(max) pH measurements (Figure S5); magnetophoretic transport of 0.20 mg BA-MNPs (Figure S6); and tabulated data showing dhyd values (Table S2) (PDF)

## Abbreviations

BA: boronic acid
MNP: magnetic nanoparticle
p*K*_a_: acid dissociation constant
GLYMO: (3-glycidyloxypropyl)trimethoxysilane
ζp: zeta potential
ISF: interstitial fluid
PDI: polydispersity index
*V*_exp_: experimental velocity
ECM: extracellular matrix
