# Supplementary material for: Surface Potential Modulation in Boronate-Functionalized Magnetic Nanoparticles Reveals Binding Interactions: Toward Magnetophoretic Capture/Quantitation of Sugars from Extracellular Matrix
Source: Langmuir. 2023 May 26;39(23):8100–8. doi: 10.1021/acs.langmuir.3c00462 (PMC10269433; doi:10.1021/acs.langmuir.3c00462)
Supplement: Supplementary file 1 — la3c00462_si_001.pdf [file la3c00462_si_001.pdf]

Surface potential modulation in boronate-functionalized magnetic nanoparticles reveals binding interactions: Towards magnetophoretic capture/quantitation of sugars from extracellular matrix

Stephen Lyons,[a] Paola Baile Pomares,[b] Lorena Vidal,[b] Katie McGarry,[c] Aoife Morrin,[a] Dermot F. Brougham[c*]*

[a]SFI Insight Centre For Data Analytics; National Centre for Sensor Research; School of Chemical Sciences, Dublin City University, Dublin 9, Ireland

[b]Departamento de Química Analítica, Nutrición y Bromatología, Instituto Universitario de Materiales, Universidad de Alicante, PO Box 99, 03080 Alicante, Spain

[c]School of Chemistry, University College Dublin, Belfield, Dublin 4, Ireland

Supplementary Information

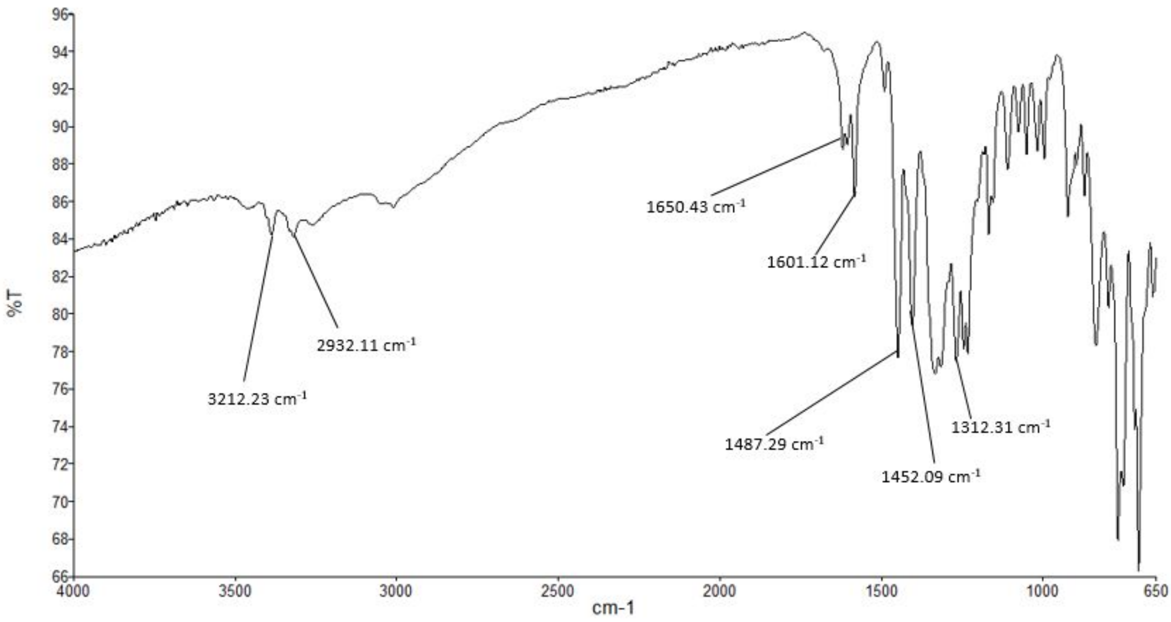

Figure S1. BA-MNP FTIR. Bands at 3212 and 2932 cm^{-1} are indicative of C-H stretches in an aromatic ring, the band at 1600 cm-1 indicate N-H bonds, when combined with the absence of bands at 1200, 950 and 800 cm^{-1}, show that the epoxy ring of the GLYMO opened and 3-aminophenylBA successfully attached via covalent bonds. Bands at 1452 cm^{-1} show the Si-O bond that indicate binding between the iron and the aminosilane of the GLYMO. The phenyl ring can be seen in the region of 1300-1500 cm^{-1}, the band at 1650 cm^{-1} shows a C=C stretch that is also present on the ring.

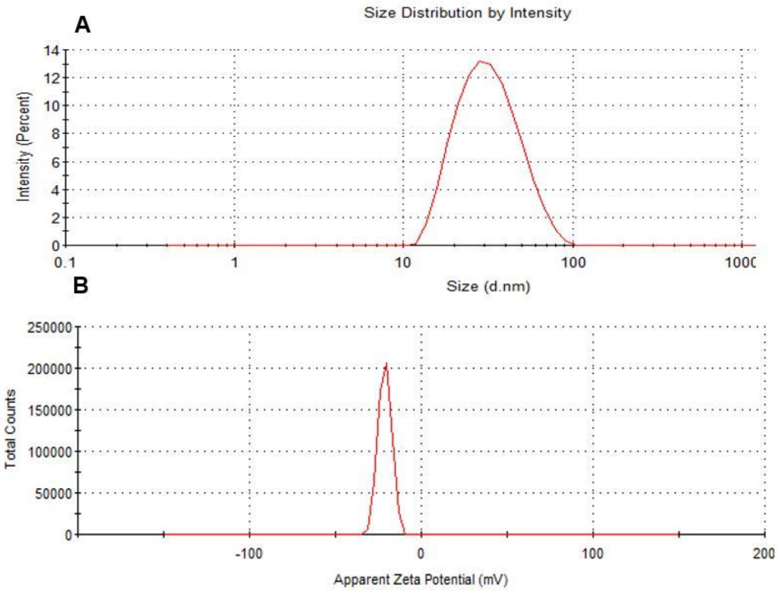

d_{hyd}:	29.5 ± 0.5
PDI	0.18 ± 0.01
ζ_p	-21 ± 0.2

Figure S2. (A) Size distribution profile of BA-MNPs and (B) ζ_p profile of BA-MNPs measured in PBS pH 7.4 (0.1 M).

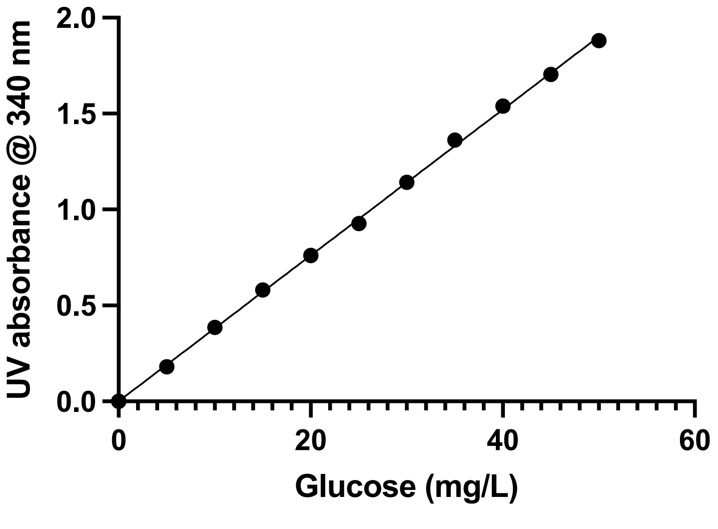

Figure S3. Absorbance measurements for glucose solutions in PBS (Total volume: 950 μl including assay reagent containing hexokinase) containing specified concentrations glucose and corresponding linear regression line (y=0.038*x + 0.0016; R^2=0.999; n=3).

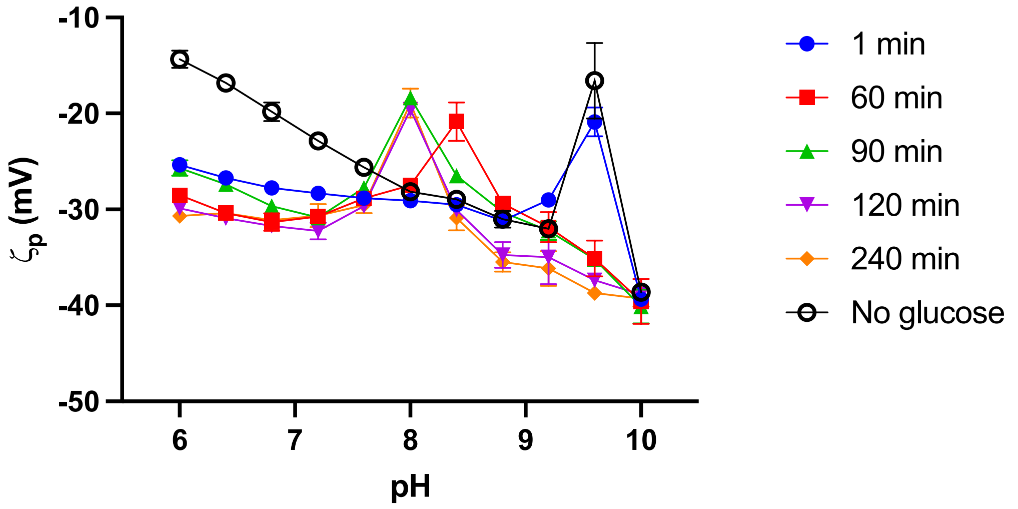

Figure S4. ζp for 2.2 mL BA-MNP suspensions (0.40 mg particles) recorded at different pH values, before incubation with glucose and at 1, 60, 90, 120 and 240 min after addition of glucose (mass 0.040 mg) see Experimental. There are ~1200:1 glucose molecules:BA-MNP present establishing MNP-limiting conditions, see text. The ionic strength was 0.10 M in all cases. Each data marker corresponds to the average and std dev values measured for 4 independent suspensions. Connecting lines are added as a visual guide.

Table S1. Tabulated ζp data for 2.2 mL BA-MNP suspensions (0.40 mg particles) recorded at different pH values, before incubation with glucose and at 1, 90 and 240 min after addition of glucose (mass 0.040 mg). Cells highlighted in green show unimodal distributions, cells highlighted in blue show bimodal distributions. ζp for the bimodal populations are given per population.

	**Zp (mV)**			
**pH**	**No glucose**	**1 min**	**90 min**	**240 min**
6	-14.4	-25.5	-25.7	-25.1
6.2	-15.1	-26.0	-26.6	-25.9
6.4	-16.8	-26.5	-27.6	-27.1
6.6	-18.7	-26.5	-28.8	-28.0
6.8	-19.8	-27.7	-29.8	-28.8
7	-22.0	-28.2	-30.7	-30.0
7.2	-22.9	-28.4	-31.1	-29.9
7.4	-24.4	-29.1	-30.2	-27.4
7.6	-25.6	-28.7	-27.5	-24.3
7.8	-27.7	-28.4	80%: -24.7; 20%: -20.1	78%: -21.1; 28%: -24.6
8	-28.2	-29.3	67%: -18.4; 33%: -17	67%: -20; 31%: -19.3, 2%: -27
8.2	-28.5	-29.5	72%: -23.6; 28%: -28.3	82%: -24.6; 18%:-25.3
8.4	-29.0	-29.8	-26.3	-27.8
8.6	-29.6	-30.0	-28.3	-30.1
8.8	-31.0	-31.1	-29.9	-30.5
9	-31.3	-27.9	-31.0	-30.6
9.2	97%: -32; 3%: -25.4	-20.3	-32.7	-
9.4	82%: -24.2; 18%: -20.3	87%: -26.5; 13%: -21.4	-34.0	-
9.6	61%: -16.6; 39%:-19.2	58%: 28.8; 42%: -20.2	-35.8	-
9.8	72%: -27.5; 28%: - 21.4	74%: -32.3; 26%: -24.3	-37.4	-
10	-38.6	-37.9	-39.7	-

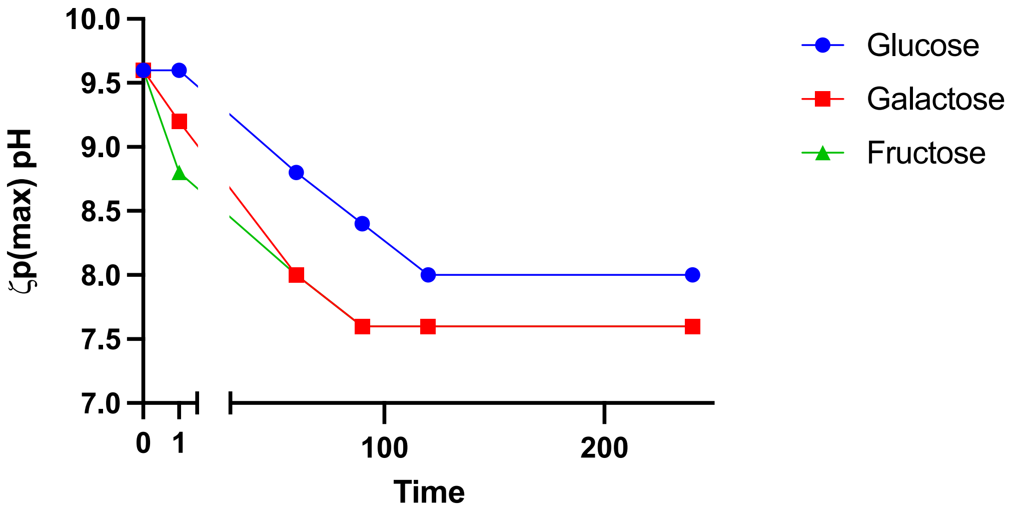

Figure S5. ζp(max) pH measurements for 2.2 mL BA-MNP suspensions (0.40 mg particles) plotted as a function of time (min) recorded after incubation with glucose (blue); galactose (red), and fructose (green) (n=4). Monosaccharide mass was 0.040 mg in all cases, giving ~600:1 sugar molecules:BA-MNP ensuring MNP-limiting conditions. Ionic strength: 0.1 M in all cases.

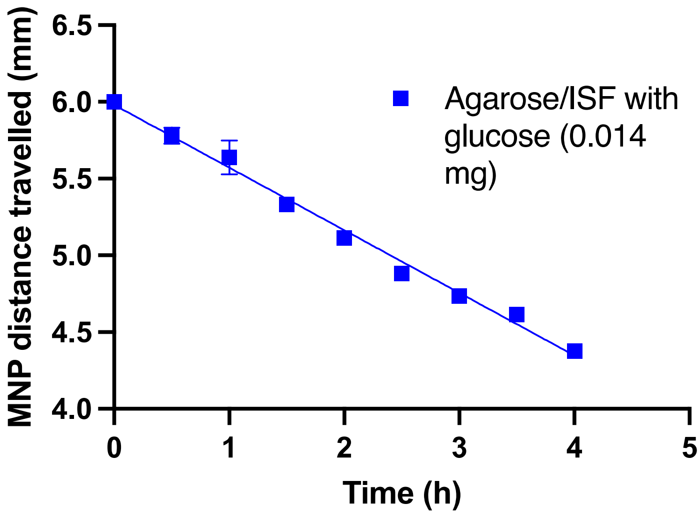

Figure S6. Magnetophoretic transport of 0.20 mg BA-MNPs, (added as 200 μL of 1.0 mg mL^{-1} BA-MNPs stock to top of gel) (d_{hyd} 29.5 nm, PDI 0.17) through 0.3% agarose/ISF containing 0.014 mg glucose. Agarose gels (700 μL; depth 6 mm). Starting mass ratio of 1:14 glucose:MNPs, glucose-limited conditions. Linear regression applied over full transit duration (y=-0.4081x+5.980), n=3.

Table S2. Tabulated data showing d_{hyd} values for BA-MNPs before and after transit through 0.3% agarose/H_2O (no glucose) and 0.3% agarose/H_2O containing 0.014 mg glucose. Agarose gels (700 μL; depth 6 mm; n=3).

**Gel matrix**	**d_{hyd} before transit (nm)**	**d_{hyd} after transit (nm)**
0.3% agarose/H_2O (no glucose)	29.5 ± 0.4	29.7 ± 0.3
0.3% agarose/H_2O (containing 0.014 mg glucose)	29.5 ± 0.4	33.2 ± 0.4
